# The Kappa Free Light Chains Index in Multiple Sclerosis: From Emerging Evidence to Clinical Application

**DOI:** 10.1111/jnc.70423

**Published:** 2026-04-01

**Authors:** Noé Verchere, Ségolène Roult, Laure Michel, Emmanuelle Le Page, Chloé Rousseau, Khaled Messaoudi, Riwan Hasbini, Claude Bendavid, Erwan Dumontet, Caroline Moreau

**Affiliations:** ^1^ Biochemistry Laboratory CHU Pontchaillou Rennes France; ^2^ Neurology CHU Pontchaillou Rennes France; ^3^ Biostatistics CHU Pontchaillou Rennes France; ^4^ Univ Rennes, CHU Rennes, INSERM, NUMECAN Rennes France; ^5^ Immunology Laboratory CHU Pontchaillou Rennes France; ^6^ Univ Rennes, CHU Rennes, INSERM, EHESP, IRSET (Institut de Recherche en Santé, Environnement et Travail) UMR_S 1085 Rennes France

**Keywords:** diagnosis algorithm, intrathecal synthesis, kappa free light chain index, multiple sclerosis

## Abstract

The kappa free light chain (KFLC) index is a promising biomarker for detecting intrathecal immunoglobulin synthesis in multiple sclerosis (MS), offering a quantitative and automated alternative to oligoclonal bands (OCBs). However, its use in clinical practice is limited by heterogeneous thresholds and a lack of standardized diagnostic algorithms. Objective to develop and validate a biological diagnostic algorithm for MS that fully integrates the KFLC‐index alongside current recommended CSF biomarkers, particularly CSF‐restricted OCBs. We conducted a prospective, monocentric study including 198 patients undergoing lumbar puncture for suspected neurological disease. Patients were classified as having MS (including CIS/RIS) or non‐inflammatory neurological disorders. Paired cerebrospinal fluid and serum KFLC and LFLC were quantified using a turbidimetric assay (Optilite, The Binding Site). Diagnostic performance was assessed by ROC analysis. A diagnostic algorithm was developed and validated in an independent cohort. In the derivation cohort (*n* = 80), the KFLC‐index showed excellent accuracy (AUC = 0.93). An optimal threshold of 20.27 provided 80.6% sensitivity and 100% specificity. A lower threshold of 3 increased sensitivity (96.8%) but reduced specificity (26.5%). Application of the diagnostic algorithm to the replication cohort (*n* = 70) confirmed the absence of false positive and false negative results for threshold values. Systematic OCBs testing enhanced interpretation in intermediate KFLC‐index ranges (3–20). The algorithm's performance was consistent with previously published thresholds. The KFLC‐index is a robust biomarker for MS diagnosis. Integration into a tiered algorithm offers excellent diagnostic performance, though local validation remains essential before broad clinical adoption.

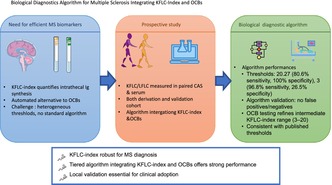

## Graphical abstract

This prospective study addresses the need for efficient multiple sclerosis (MS) biomarkers by validating a biological diagnostic algorithm. Based on paired cerebrospinal fluid (CSF) and serum samples from derivation and validation cohorts, the algorithm integrates the kappa free light chain (KFLC) index and oligoclonal bands (OCBs). A KFLC‐index > 20.27 provides 100% specificity for MS, while values < 3 exclude the diagnosis with 96.8% sensitivity. OCB testing is reserved to refine the intermediate range (3–20), optimizing laboratory workflow. This robust, automated alternative demonstrates strong diagnostic performance and supports standardized clinical adoption.

**Abbreviations**
AUC: area under the curve
CI: confidence interval
CIS: clinically isolated syndrome
CNS: central nervous system
CSF: cerebrospinal fluid
CVS: central vein sign
DC: derivation cohort
F: fisher's exact test
FLC: free light chain
Ig: immunoglobulin
IgG‐index: immunoglobulin G index
IQR: interquartile range
K: chi‐squared test
KFLC: kappa free light chain
LFLC: lambda free light chain
LLOQ: lower limit of quantification
MOGAD: myelin oligodendrocyte glycoprotein antibody‐associated disease
MS: multiple sclerosis
MWU: Mann–Whitney–Wilcoxon tests
N: number of observations
NIND: non‐inflammatory neurological disorders
NMOSD: neuromyelitis optica spectrum disorder
NPV: negative predictive value
OCB: oligoclonal bands
OIND: other inflammatory CNS disorders
PIRA: progression independent of relapse activity
PPV: positive predictive value
QAlb: albumin quotient (CSF/serum)
QKFLC: KFLC quotient (CSF/serum)
QLFLC: LFLC quotient (CSF/serum)
RC: replication cohort
RIS: radiologically isolated syndrome
ROC: receiver operating characteristic
RRID: Research Resource Identifier (see scicrunch.org)
RRMS: relapsing–remitting multiple sclerosis
S: Student's *t*‐test
SD: standard deviation
W: Mann–Whitney‐Wilcoxon test

## 1 Introduction

Multiple sclerosis (MS) is a chronic autoimmune disorder of the central nervous system (CNS), characterized by inflammation, demyelination, and axonal damage. Clinical presentation varies but often includes optic neuritis, myelitis, sensory symptoms, or brainstem syndromes (MP et al. 2021). MS commonly affects young adults, with a female predominance (Dobson and Giovannoni 2019). Diagnosis relies on demonstrating dissemination in time and space, using clinical, radiological, and laboratory data. Detection of intrathecal immunoglobulin (Ig) synthesis is central to MS diagnosis. While cerebrospinal fluid (CSF)‐restricted oligoclonal bands (OCBs) remain the reference method (Freedman et al. 2005), this technique is qualitative, labor‐intensive, and operator‐dependent. The IgG‐index, though automated, lacks sufficient sensitivity and is no longer considered reliable on its own (Thompson et al. 2018; Link and Huang 2006). The revised 2024 McDonald criteria (Thompson et al. 2018) enhance diagnostic sensitivity by introducing new biomarkers and topographies, and the kappa free light chain (KFLC) index is now incorporated as an alternative to OCBs to evidence intrathecal immunoglobulin synthesis (Deisenhammer et al. 2025). Indeed, the KFLC‐index has recently emerged as a promising quantitative alternative. Calculated from paired CSF and serum samples, the KFLC‐index is reproducible, automated, and cost‐effective, and may outperform OCBs in early or OCB‐negative disease (Levraut, Gavoille, et al. 2023; Leurs et al. 2020; Hegen, Walde, et al. 2023; Hegen, Arrambide, et al. 2023; Beckmann et al. 2025). Despite extensive validation, the absence of standardized thresholds limits routine clinical adoption (Ferraro et al. 2020; Levraut, Laurent‐Chabalier, et al. 2023; Senel et al. 2019; Menéndez‐Valladares et al. 2019; Gaetani et al. 2020; Bernardi et al. 2022). Importantly, the 2024 McDonald criteria revision now formally includes the KFLC‐index as an accepted marker of intrathecal synthesis, alongside OCBs (Deisenhammer et al. 2025; Levraut et al. 2025).

Beyond its diagnostic role, the KFLC‐index also provides prognostic value. Elevated KFLC‐index has been shown to predict conversion to MS in patients with radiologically isolated syndrome (RIS) or clinically isolated syndrome (CIS), correlate with early disease activity, and is associated with higher relapse risk, progression independent of relapse activity (PIRA), and cognitive decline (Rosenstein, Axelsson, Novakova, Malmeström, et al. 2023; Rosenstein, Axelsson, Novakova, Rasch, et al. 2023). Increased baseline KFLC‐index predicts a second demyelinating event and development of new T2 lesions, even independently of other biomarkers (Levraut, Gavoille, et al. 2023; Vecchio et al. 2020; Moreno‐Navarro et al. 2025). These findings further reinforce the rationale for incorporating the KFLC‐index into clinical algorithms for MS diagnosis and prognostication.

In this context, the objective of this study is to develop and validate a biological diagnostic algorithm for multiple sclerosis that fully integrates the KFLC‐index into the panel of currently recommended diagnostic tools, notably the detection of intrathecal immunoglobulin synthesis by CSF‐restricted OCBs. This work is explicitly aligned with the 2024 revision of the McDonald criteria, which now include the KFLC‐index as CSF biomarkers for intrathecal Ig synthesis. Prioritizing specificity, our approach aims to support confident rule‐in decisions, address diagnostic uncertainty in early or atypical presentations, and promote reliable early identification of MS to facilitate optimal patient care. To accomplish this, two independent cohorts were established: a derivation cohort to develop a biological diagnostic algorithm, and a replication cohort to validate its diagnostic performance.

## 2 Patients and Methods

### 2.1 Patients and Controls

The research protocol was conducted in accordance with French legal and ethical guidelines and was approved by the Research and Innovation Department of Rennes University Hospital. This monocentric, non‐interventional study falls outside the scope of the Jardé Law, complies with the MR‐004 reference methodology, and involves no data transfer outside the institution. The study was approved by the Ethics Committee of Rennes University Hospital in accordance with applicable ethical standards (approval number: 2025‐205).

Patients were prospectively enrolled if they underwent a lumbar puncture with a prescription for OCBs testing at Rennes University Hospital for routine analysis. After completion of the OCBs analysis, the remaining CSF and serum samples were stored at −20 C. Eligible patients received an information letter. In the absence of any objection within 3 weeks of mailing, kappa and lambda free light chain (KFLC and LFLC) concentration and indexes were measured.

**FIGURE 1:**
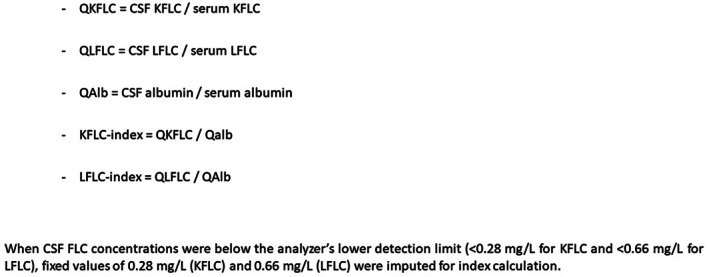
Various formulae used to assess the KFLC‐ and LFLC indexes. CSF, cerebrospinal fluid; FLC, free light chain; QALB, CSF/serum albumin quotient; QKFLC, KFLC quotient; QLFLC, LFLC quotient.

Inclusion took place from November 2023 to June 2024 for the derivation cohort, and from August 2024 to March 2025 for the replication cohort. No a priori sample size calculation was performed; instead, a prospective and consecutive inclusion strategy was adopted over these fixed periods to reflect the real‐world clinical flow of the department and to maximize the representativeness of the cohorts. Following this “real‐world” prospective inclusion, a total of 94 and 115 patients were initially captured in the derivation cohort and in the replication cohort, respectively. To ensure a rigorous evaluation of the KFLC‐index for MS diagnosis, we strictly excluded patients diagnosed with other inflammatory neurological diseases (OIND), such as Neuromyelitis optica spectrum disorder (NMOSD), Myelin oligodendrocyte glycoprotein antibody‐associated disease (MOGAD), neurosarcoidosis, or CNS infections, as these conditions are known to potentially involve intrathecal immunoglobulin synthesis. In each independent cohort, the remaining patients were divided into two groups based on the final diagnosis established by the clinician, using clinical, biological, and imaging data in accordance with the 2017 McDonald criteria (Thompson et al. 2018): the MS group (including patients with MS, CIS, and RIS) (Dobson and Giovannoni 2019), and the non‐inflammatory neurological disorders group (NIND; primarily neurodegenerative diseases). A detailed breakdown of the NIND group and the exclusion process is provided in Figure 1 and in the Supporting Information (Table S2). Diagnostic data were extracted from patients' medical records.

### 2.2 Laboratory Features

OCBs were assessed by isoelectric focusing followed by immunodetection using a labeled IgG‐specific antibody, according to the manufacturer's instructions (Hydragel 9 CSF Isofocusing kit, Sebia, Lisses, France; Cat. No. 4355) on a Hydrasys 2 Scan Focusing system (Sebia, Cat. No. 1202). OCBs patterns were independently evaluated by two experienced biologists, in accordance with current guidelines (Gastaldi et al. 2017).

For routine analyses related to OCBs interpretation, CSF albumin and IgG concentrations were measured using nephelometry on the BNII analyzer (Siemens Healthcare Diagnostics, Marburg, Germany; REF OVIA11, SMN 11254820) using the N Antisera to Human IgG (Cat. No. OSAS09, 10 446 296) and N Antisera to Human Albumin (Cat. No. OSAL15, 10 446 283). Serum IgG and albumin were measured on a Cobas platform (Roche Diagnostics, Basel, Switzerland; RRID: SCR_001326) using the IGG2 Tina‐quant IgG Gen.2 (Cat. No. 08057915190) and the Albumin Gen.2 bromocresol green method (Cat. No. 05166861188). Method comparison studies performed in‐house showed good agreement between these techniques, supporting their combined use in routine diagnostic workflows. The albumin quotient (QAlb) (Figure 1) serves as a marker of blood–CSF barrier integrity. Normal reference values for QAlb increase with age, reflecting physiological changes in barrier permeability over the lifespan. The IgG‐index is calculated as the ratio of CSF‐to‐serum IgG, normalized to the CSF‐to‐serum albumin ratio. It reflects intrathecal IgG synthesis independently of blood–CSF barrier function. Normal values are < 0.7, although slight variations may occur depending on age.

For the calculation of KFLC‐ and LFLC‐ indexes, CSF and serum levels of albumin, KFLC, and LFLC were all measured using Freelite assays (The Binding Site Group Ltd., Birmingham, UK; KFLC: Cat. No. LK016.OPT, LFLC: Cat. No. LK017.OPT, RRID:SCR_004051) on an Optilite turbidimeter (The Binding Site Group Ltd., RRID:SCR_004051), in accordance with the manufacturer's instructions. This analytical consistency ensures the reliability of the calculated indexes. Various formulae are presented in Figure 1.

### 2.3 Statistical Analysis

Quantitative variables are described using the following metrics: number of observations (*N*), mean ± standard deviation (SD), minimum, first quartile (Q1), median, third quartile (Q3), and maximum. Normality of the data was assessed using both visual inspection with Q‐Q plots and the Shapiro–Wilk test. For normally distributed continuous variables, two‐tailed parametric Student's *t*‐tests (S) were used, and results are presented as mean ± SD. For non‐normally distributed variables, two‐tailed non‐parametric Mann–Whitney–Wilcoxon tests (MWU) were applied, and results are presented as median (Q1–Q3). No formal test for outliers was performed, and no data points were excluded; however, the use of non‐parametric tests when normality was not met helped reduce the impact of extreme values. For qualitative variables, the number (*N*) and percentage (%) were reported for each category. Group comparisons were performed using either a chi‐squared test or Fisher's exact test (*F*), as appropriate.

The diagnostic performances of the KFLC‐ and LFLC‐indexes were evaluated using receiver operating characteristic (ROC) curve analysis, with calculation of the area under the curve (AUC). A paired sample area difference under the ROC curves analysis was used to compare the different ROC curves. ROC curves were devised for KFLC‐index and LFLC‐index, and the Youden index was used to identify optimal cutoff values for KFLC‐ and LFLC‐indexes.

For all analyses, *p*‐values < 0.05 were considered statistically significant.

Statistical analyses were performed using SAS software, version 9.4.

## 3 Results

### 3.1 Demographic and Clinical Data in Derivation Cohort

**FIGURE 2:**
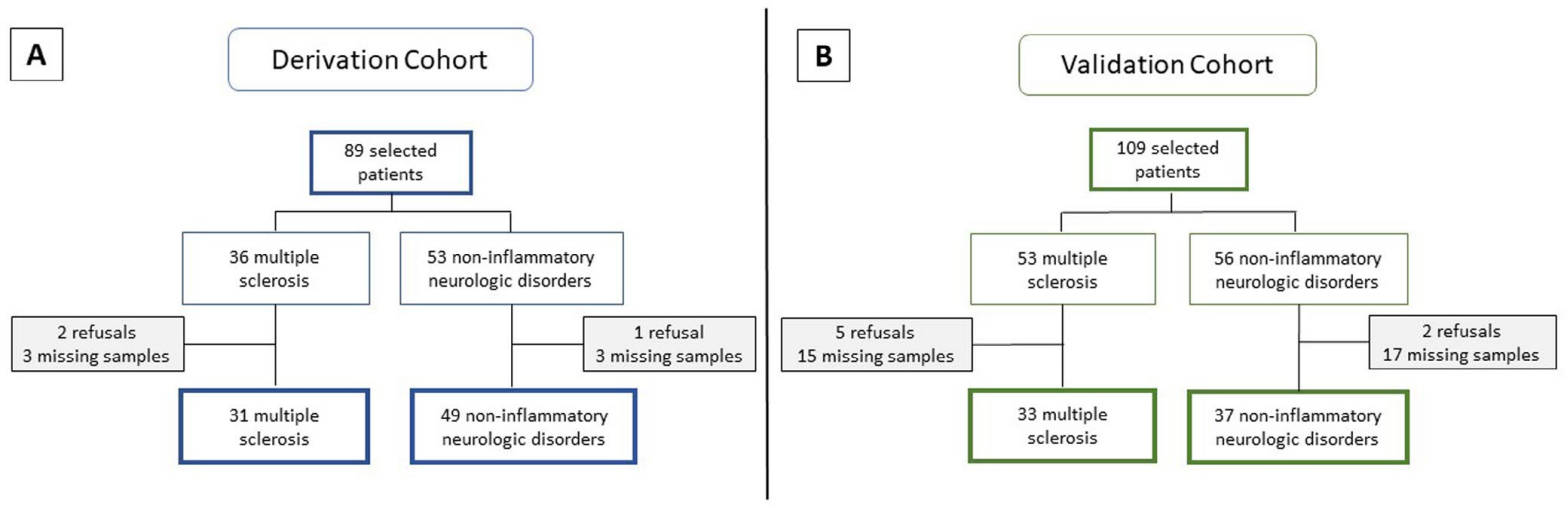
Flow chart of the patients' inclusion process in the Derivation cohort (A) and in the Replication cohort (B). CNS, central nervous system.

Eighty‐nine patients were initially selected from November 2023 to June 2024 for study in the derivation cohort. Paired CSF and serum samples were available for 83 patients. Three patients declined to participate in the study. Thirty‐one patients were included in the MS group, which consisted of 20 patients with RRMS, 5 with progressive MS phenotypes, 5 with RIS, and 1 with CIS, and 49 in the NIND group (Tables S1 and S2, Figure 2A). Detailed clinical characteristics and a comprehensive list of diagnoses for the non‐inflammatory neurological disorders (NIND) group—which primarily included neurodegenerative, vascular, and epileptic disorders—are provided in Table S2. Demographic and clinical data at the time of serum/CSF sampling are presented in Table 1. Characteristics that differed significantly between the MS and NIND groups included age (median 40 vs. 61 years, respectively; *p* = 0.0002), CSF protein concentration (median 0.4 vs. 0.3 g/L; *p* = 0.0336), blood‐CSF barrier dysfunction prevalence (19.4% vs. 0%; *p* = 0.0025), CSF IgG levels (median 47 mg/L vs. 25 mg/L; *p* < 0.0001), IgG‐index values (median 0.8 vs. 0.4; *p* < 0.0001), and OCBs positivity (90.3% vs. 0%; *p* < 0.0001).

**TABLE 1:** Demographic and clinical data at serum/CSF sampling in the derivation cohort.

	MS (*n* = 31)	NIND (*n* = 49)	Statistic test	*p*
Age (years), median [IQR]	40 [25–48]	61 [39–69]	MWU U = 884	**0.0002**
Sex (women), *n* (%)	24 (77.4)	34 (69.4)	K *χ* ^2^(1) = 0.61	0.4332
CSF protein (g/L), median [IQR]	0.4 [0.3–0.5]	0.3 [0.3–0.4]	S *t*(78) = 2.16	**0.0336**
Serum protein (g/L), median [IQR]	70 [67–73]	69 [66–73]	S *t*(78) = 2.16	0.7200
CSF albumin (mg/L), median [IQR]	234 [176–286]	220 [164–258]	S *t*(78) = 2.16	0.2676
Serum albumin (g/L), median [IQR]	41.5 [38.6–43.0]	41.6 [39.9–44.4]	MWU U = 1194.5	0.5501
Albumin quotient, median [IQR]	0.5 [0.4–0.7]	0.5 [0.4–0.7]	MWU U = 1316.5	0.5502
Blood CSF barrier dysfunction, *n* (%)	6 (19.4)	0 (0.0)	F	**0.0025**
CSF IgG (mg/L), median [IQR]	47.0 [30.0–64.0]	25.0 [18.0–31.0]	MWU U = 1791.5	**< 0.0001**
Serum IgG (g/L), median [IQR]	10.6 [9.7–14.0]	10.6 [8.9–12.8]	S *t*(78) = 2.16	0.4678
IgG‐index, median [IQR]	0.8 [0.5–1.0]	0.4 [0.4–0.5]	MWU U = 1917.5	**< 0.0001**
Positive OCBs, *n* (%)	28 (90.3)	0 (0.0)	K *χ* ^2^(1) = 54	**< 0.0001**
CSF KFLC concentration (mg/L), median [IQR]	4.3 [1.1–9.1]	0.3 [0.3–0.3]	MWU U = 1988	**< 0.0001**
Serum KFLC concentration (mg/L), median [IQR]	12.2 [9.4–13.8]	13.1 [10.3–16.3]	MWU U = 1139	0.2520
KFLC‐index, median [IQR]	64.9 [25.6–149.4]	3.9 [2.9–6.2]	MWU U = 1910	**< 0.0001**
CSF LFLC concentration (mg/L), median [IQR]	1.2 [0.9–2.4]	0.7 [0.7–0.8]	MWU U = 1899	**< 0.0001**
Serum LFLC concentration (mg/L), median [IQR]	11.9 [8.8–14.8]	12.7 [10.0–15.9]	MWU U = 1168	0.3902
LFLC‐index, median [IQR]	22.0 [12.6–53.2]	10.1 [7.7–20.9]	MWU U = 1636	**0.0002**

*Note:* Bold indicates statistically significant.

Abbreviations: CSF, cerebrospinal fluid; IQR, interquartile range; *K*, chi squared test; MS, multiple sclerosis; MWU, Mann–Whitney Wilcoxon test; NIND, non‐inflammatory neurologic disorders; OCBs, oligoclonal bands; S, Student's *t*‐test; *F*, fisher's test.

### 3.2 Diagnostic Performance of KFLC‐ and LFLC‐Indexes for Development of a Pragmatic Biological Diagnostic Algorithm

**FIGURE 3:**
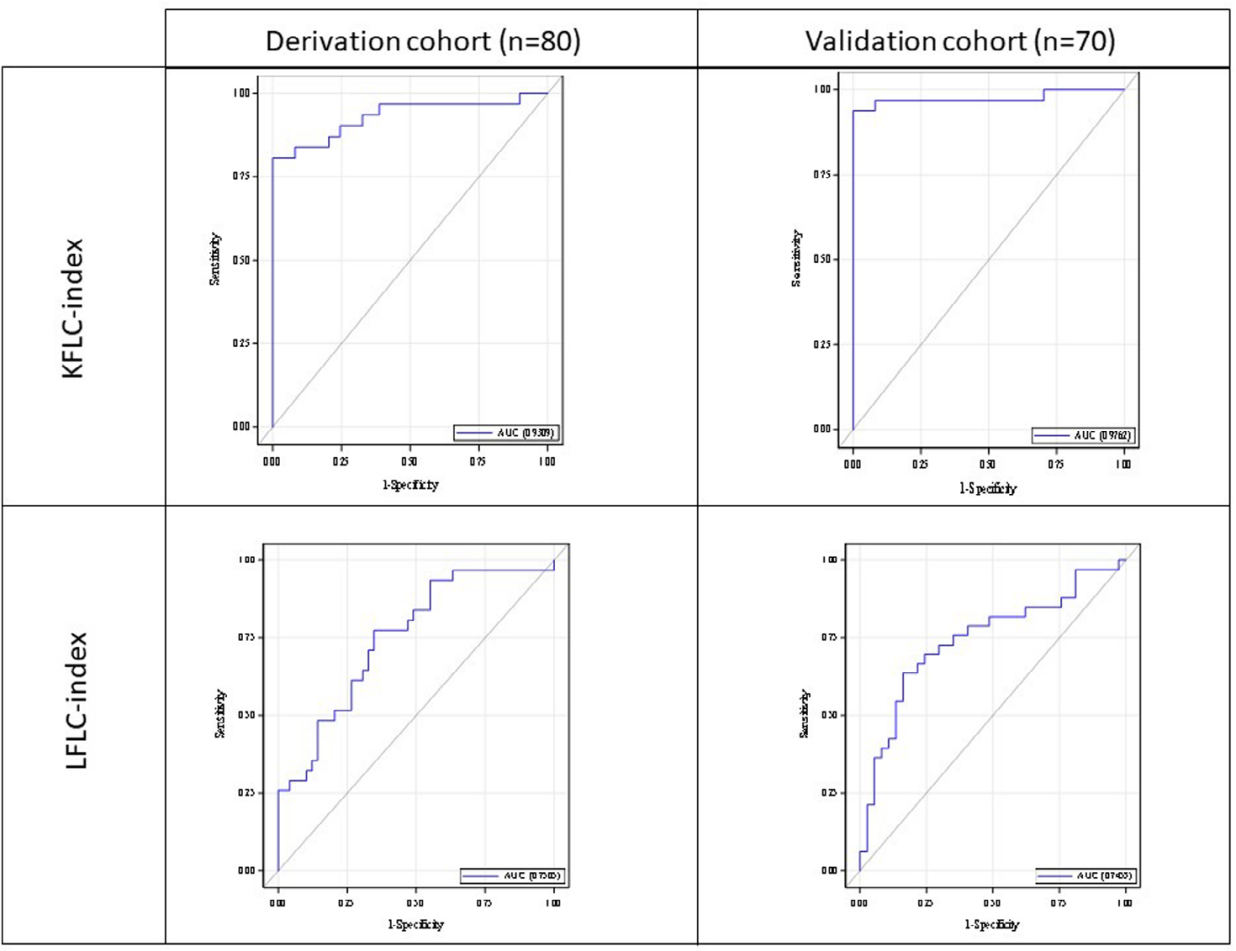
ROC curves for KFLC index and LFLC index to detect Multiple Sclerosis in control patients in the Derivation cohort and in the Replication cohort.

The diagnostic performance of KFLC‐ and LFLC‐indexes to identify patients with MS was assessed using ROC curve analysis. In the derivation cohort, the KFLC‐index outperformed the LFLC‐index, with a significantly higher AUC (0.931 vs. 0.751, *p* = 0.0001) when discriminating MS from NIND (Figure 3). A KFLC‐index threshold of 20.27 provided a specificity of 100% (95% CI [100%–100%]) and a sensitivity of 80.7% (95% CI [66.7%–94.6%]) (Table 2, Figure 3). This cut‐off correctly classified all NIND patients and 25 out of 31 MS patients. Among the six MS patients with a KFLC‐index below 20.27, three had relapsing–remitting MS, including two OCBs‐positive individuals (KFLC‐index: 8.24 and 10.47) and one 81‐year‐old woman with longstanding, inactive disease (KFLC‐index: 2.53, OCBs‐negative). One patient had a CIS (KFLC‐index: 4.82, OCBs‐positive), while two had RIS (KFLC‐index: 6.94 and 5.14), both OCBs‐negative.

**TABLE 2:** Diagnostic performance of KFLC‐ and LFLC‐indexes for differentiating MS from NIND patients in the derivation (DC) and replication (RC) cohorts.

Threshold		Sensitivity [95% CI]	Specificity [95% CI]	PPV [95% CI]	NPV [95% CI]
KFLC‐index ≥ 20.27	DC	80.65% [66.7–94.6]	100% [100–100]	100% [100–100]	89.09% [80.9–97.3]
	RC	75.76% [64.1–90.4]	100% [100–100]	100% [100–100]	82.22 [71.1–93.4]
KFLC‐index ≥ 3	DC	96.77% [90.6–100]	26.53% [14.2–38.9]	45.45% [33.4–57.5]	92.86% [79.4–100]
	RC	100% [100–100]	18.92 [6.3–31.5]	52.38% [40.6–64.7]	100% [100–100]
LFLC‐index ≥ 12.61	DC	77.42% [62.7–92.1]	65.31% [52–78.6]	58.54% [43.5–73.6]	82.05% [70–94.1]
	RC	84.85% [72.6–97.1]	32.43% [17.3–47.5]	52.83% [39.4–66.3]	70.59% [48.9–92.2]
LFLC‐index ≥ 7	DC	96.77% [90.6–100]	16.33% [6–26.7]	42.25% [30.8–53.7]	88.89% [68.4–100]
	RC	96.97% [91.1–100]	5.41% [1.9–12.7]	47.76% [35.8–59.7]	66.67% [13.3–100]

Lowering the KFLC‐index threshold to 3 resulted in a sensitivity of 96.77% (95% CI [90.3%–100%]) and a specificity of 26.53% (95% CI [14.2%–38.9%]). At this threshold, only the previously mentioned elderly patient with inactive disease remained below the cut‐off. Detailed results on the KFLC‐ and LFLC‐indexes are provided in Table 2, Figure 3.

**FIGURE 4:**
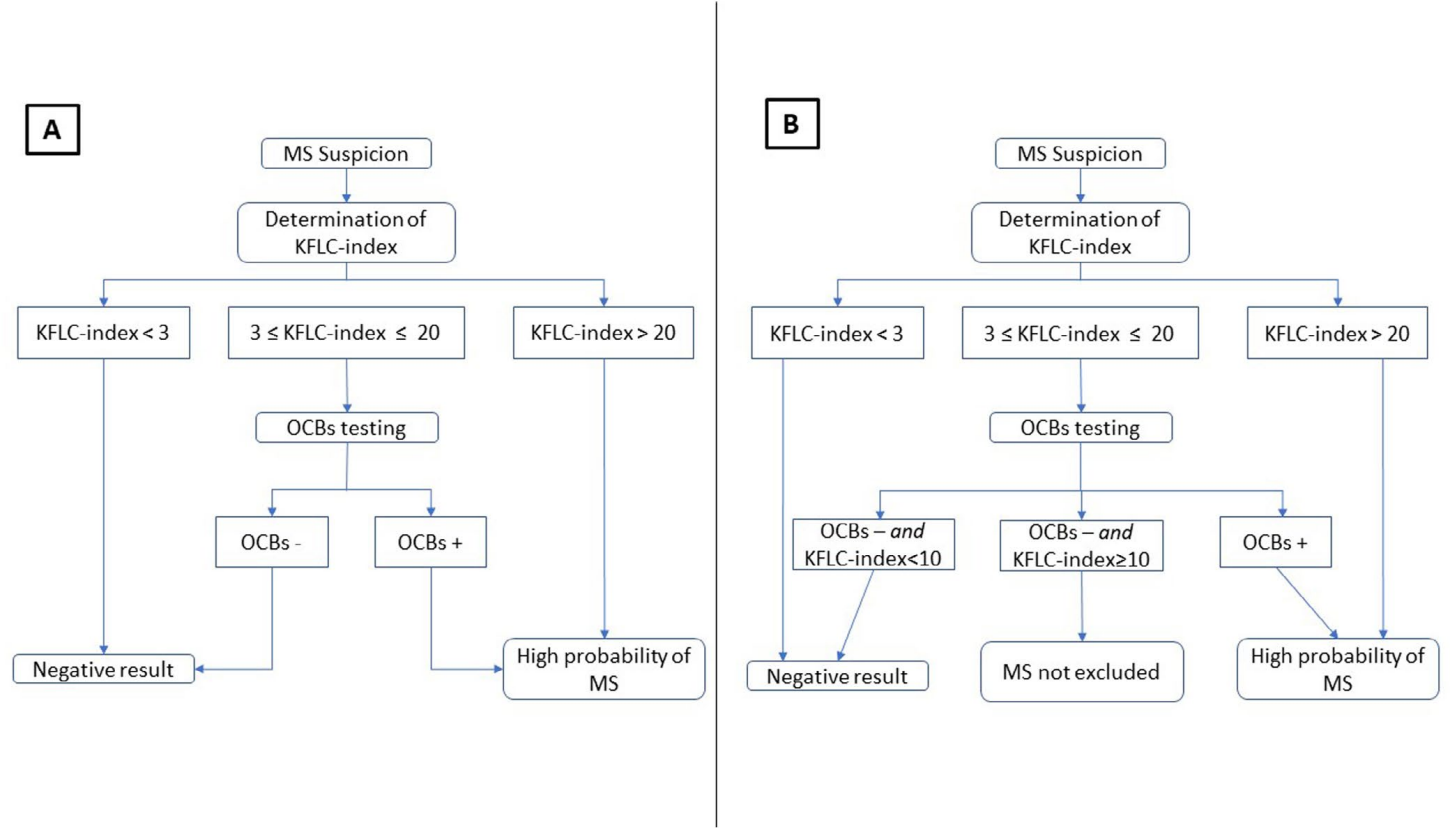
Proposed biological diagnostic algorithms for multiple sclerosis (MS) based on KFLC‐index thresholds and oligoclonal band (OCB) testing. (A) Algorithm derived from the derivation cohort data utilizing both KFLC‐index and CSF‐restricted OCBs for robust identification of MS cases. A KFLC‐index below 3 consistently indicates a very low probability of MS, allowing early exclusion and reduction of unnecessary CSF analyses. Values above 20, rounded from the optimal 20.27 to improve clinical practicality, provide high specificity for MS diagnosis, even in cases without positive OCBs. For intermediate KFLC‐index values (3–20), systematic OCB testing refines diagnostic stratification: OCB positivity maintains significant discriminatory power, ensuring cases are not missed. (B) Algorithm adapted to incorporate a cut‐off of 10 within the intermediate KFLC‐index range (3–20). KFLC‐index values below 3 suggest low MS probability, potentially avoiding further CSF workup; values above 20 support early diagnosis with high confidence; intermediate values require OCB testing, where a negative OCB result combined with KFLC‐index below 10 indicates a negative diagnosis, whereas KFLC‐index ≥ 10 with negative OCBs does not exclude MS; OCB positivity regardless of KFLC‐index in this range strongly suggests MS.

Based on these thresholds, a biological decision algorithm was structured (Figure 4). The threshold of 3 was selected to maximize sensitivity, as patients with a KFLC‐index below this value consistently showed a very low probability of MS. Conversely, a KFLC‐index threshold of 20 was retained to ensure optimal specificity. This value was rounded from the calculated 20.27 for greater clinical practicality and ease of use in routine practice. Patients are thus categorized into three groups: KFLC‐index < 3, KFLC‐index between 3 and 20, and KFLC‐index > 20. In the intermediate range (3–20), the status of intrathecal synthesis is determined by OCB testing, which provided the necessary discriminatory power to classify cases that fell below the high‐specificity threshold.

### 3.3 Replication Cohort Analysis

To confirm our findings, we analyzed an independent replication cohort including 109 consecutively selected patients between August 2024 and March 2025. After exclusion of patients who declined to participate (*n* = 7) or had missing samples (*n* = 32), finally, 70 patients were included for analysis (Figure 2B). Among them, 33 patients had a confirmed diagnosis of MS with 26 RRMS, 2 progressive MS (including 1 Marburg variant), 4 RIS, and 1 CIS, and 37 had NIND and were classified as controls. The same inclusion criteria and analytical procedures as in the derivation cohort were applied. Demographic and clinical data at the time of serum/CSF sampling are presented in Table 3.

**TABLE 3:** Demographic and clinical data at serum/CSF sampling in the replication cohort.

	MS (*n* = 33)	NIND (*n* = 37)	Statistic test	*p*
Age (years), median [IQR]	31.2 [27.7–43.5]	55.3 [47.1–59.9]	MWU U = 784	**< 0.0001**
Sex (women), *n* (%)	24 (72.7)	22 (59.5)	K *χ* ^2^(1) = 1.36	0.2430
CSF protein (g/L), median [IQR]	0.4 [0.3–0.4]	0.4 [0.4–0.4]	MWU U = 1270	0.2484
Serum protein (g/L), median [IQR]	70 [68–73]	67 [65–71]	MWU U = 1361	0.0256
CSF albumin (mg/L), median [IQR]	230.7 [197.8–283.6]	219.5 [190.1–270.2]	MWU U = 1236.5	0.4479
Serum albumin (g/L), median [IQR]	46.2 [43.7–48.3]	44.3 [42.1–46.3]	MWU U = 1405	0.0061
Albumin quotient, median [IQR]	0.5 [0.4–0.6]	0.6 [0.4–0.6]	MWU U = 1129	0.6212
Blood CSF barrier dysfunction, *n* (%)	6 (18.8)	0 (0.0)	F	**0.0046**
CSF IgG (mg/L), median [IQR]	37.0 [28–58]	21 [17–29]	MWU U = 1558.5	**< 0.0001**
Serum IgG (g/L), median [IQR]	11.7 [10.3–13.0]	10.0 [8.5–11.3]	MWU U = 1372	**0.0186**
IgG‐index, median [IQR]	0.63 [0.5–1.1]	0.43 [0.4–0.5]	MWU U = 1732.5	**< 0.0001**
Positive OCBs, *n* (%)	30 (90.9)	1 (2.7)	K *χ* ^2^(1) = 55	**< 0.0001**
CSF KFLC (mg/L), median [IQR]	3.31 [1.3–4.1]	0.28 [0.3–0.3]	MWU U = 1775	**< 0.0001**
Serum KFLC (mg/L), median [IQR]	10.9 [10.1–14.6]	11.6 [10.4–16.2]	MWU U = 1115	0.5100
KFLC‐index, median [IQR]	45.9 [21.0–89.4]	4.9 [3.2–6.5]	MWU U = 1753	**< 0.0001**
CSF LFLC (mg/L), median [IQR]	1.59 [1.1–2.0]	0.9 [0.8–1.0]	MWU U = 1654.5	**< 0.0001**
Serum LFLC (mg/L), median [IQR]	11.2 [9.5–12.5]	10.6 [8.6–14.6]	MWU U = 1202	0.7241
LFLC‐index, median [IQR]	30.25 [20.5–43.5]	18.2 [12.3–23.8]	MWU U = 1471	**0.0004**

*Note:* Bold indicates statistically significant.

Abbreviations: CSF, cerebrospinal fluid; F, fisher's test; IQR, interquartile range; K, chi squared test; MS, multiple sclerosis; MWU, Mann–Whitney Wilcoxon test; NIND, non‐inflammatory neurologic disorders; OCBs, oligoclonal bands; S, Student's *t*‐test.

Biological characteristics were overall similar between the derivation and replication cohorts (Tables 1 and 3), and between the two MS cohorts, as well as between the two NIND control groups (Tables 1 and 2). In accordance with CONSORT recommendations, no formal statistical testing was performed between cohorts, as these data are presented solely for descriptive purposes (Supporting Information: Tables S1 and S2). Among MS patients, median CSF KFLC concentrations and the KFLC‐index were slightly lower in the replication cohort (3.3 mg/L [1.3–4.1] vs. 4.3 mg/L [1.1–9.1], and 45.9 [21.0–89.4] vs. 64.9 [25.6–149.4], respectively). Conversely, CSF LFLC concentration and the LFLC‐index were slightly higher (1.6 mg/L [1.1–2.0] vs. 1.2 mg/L [0.9–2.4]; 30.3 [20.5–43.5] vs. 22.0 [12.6–53.2]). In NIND controls, CSF KFLC and LFLC concentrations were comparable, but both KFLC‐ and LFLC‐indexes were higher in the replication cohort (4.9 [3.2–6.5] vs. 3.9 [2.9–6.2]; 18.2 [12.3–23.8] vs. 10.1 [7.7–20.9]).

In the derivation cohort, the optimal KFLC‐index threshold was 10.82, with a sensitivity of 93.9% (95% CI [85.8%–100%]) and specificity of 100% (95% CI [100%–100%]), with corresponding PPV and NPV of 100% (95% CI [85.8%–100%]) and 94.9% (95% CI [87.9%–100%]), respectively. Applying the pre‐defined threshold of 20.27 to this cohort maintained a specificity of 100% (95% CI [100%–100%]) with a sensitivity of 75.8% (95% CI [64.1%–90.4%]) (Table 4).

**TABLE 4:** Diagnostic performance of KFLC‐ and LFLC‐indexes for differentiating MS from NIND patients in the replication cohorts.

Threshold	Sensitivity [95% CI]	Specificity [95% CI]	PPV [95% CI]	NPV [95% CI]
KFLC‐index ≥ 10.82	93.94% [85.8–100]	100% [100–100]	100% [100–100]	94.87 [87.9–100]
LFLC‐index ≥ 26.79	63.64% [47.2–80]	83.78% [71.9–95.7]	77.78% [62.11–93.5]	72.09% [58.7–85.5]

In this cohort, the LFLC‐index threshold of 26.79 achieved a sensitivity of 63.6% (95% CI [47.2%–80%]) and specificity of 83.8% (95% CI [71.9%–95.7%]), with corresponding PPV and NPV of 77.8% (95% CI [62.1%–93.5%]) and 72.1% (95% CI [58.7%–85.5%]), respectively. The LFLC‐index threshold of 12.61 produced a sensitivity of 84.9% (95% CI [72.6%–97.1%]) and a specificity of 32.4% (95% CI [17.3%–47.5%]) (Table 2, Figure 3).

When the three‐tier algorithm was applied to the replication cohort, all patients with a KFLC‐index < 3 were NIND (*n* = 7), and all those with a KFLC‐index > 20 were MS (*n* = 25). Within the intermediate range (3–20), patients should have benefited from OCBs testing. In this range, one 32‐year‐old man with clinically confirmed RRMS, had a KFLC‐index of 8.16 (below the threshold of 20.27), but was OCBs‐positive (type 2 pattern), with no blood–brain barrier disruption (albumin ratio: 0.46) and a normal CSF total protein level (0.3 g/L), allowing for correct classification through the combined approach.

Overall, the implementation of this algorithm would have obviated the need for OCBs testing in 47.5% (38/80) of the derivation cohort and 45.7% (32/70) of the replication cohort. These results demonstrate that a high‐stringency rule‐in threshold significantly streamlines laboratory workflows without compromising diagnostic safety, effectively reserving OCB testing for the most biochemically ambiguous cases.

## 4 Discussion

The importance of combining biological, radiological, and clinical markers in the diagnostic process of MS is now widely acknowledged. According to the 2017 McDonald criteria revised by Thompson et al. (2018), the diagnosis of MS is based on demonstrating dissemination in time and space through clinical relapses, MRI findings, and biological evidence of intrathecal IgG synthesis. OCBs, long considered the gold standard biomarker of intrathecal inflammation, were formally integrated into diagnostic criteria in 2017 as a substitute for demonstrating dissemination in time in patients presenting with a typical clinically isolated syndrome and MRI evidence of dissemination in space. However, OCBs detection is a qualitative and labor‐intensive method, subject to inter‐operator variability, and requiring expert interpretation.

The 2024 McDonald updated criteria introduce two major advances: first, the recognition of the KFLC‐index as a quantitative, automated measure of intrathecal immunoglobulin production equivalent to OCBs, and second, the integration of novel radiological markers including the central vein sign (CVS) and paramagnetic RIM lesions, both highly specific for perivenular demyelination in MS (Deisenhammer et al. 2025; Levraut et al. 2025). Optic nerve involvement is now formally accepted as a fifth topographic region fulfilling dissemination in space, and RIS can be diagnosed as MS if CSF or imaging criteria are met without waiting for clinical symptoms. Furthermore, the requirement for dissemination in time has been relaxed, permitting faster and earlier diagnosis, particularly in atypical and pediatric cases. These additions substantially enhance the sensitivity and specificity of the diagnostic process.

In this study, we evaluated the diagnostic usefulness of the KFLC‐index for MS by developing and validating a pragmatic, threshold‐based biological decision algorithm. Using clinically relevant cut‐offs (< 3 to rule out MS, > 20 to rule in MS, rounded for practicality from 20.27), we achieved high diagnostic performance in both the derivation and replication cohorts, consistent with accumulating evidence that supports the KFLC‐index as a robust biomarker for intrathecal immunoglobulin synthesis. The choice of a high rule‐in threshold (> 20) prioritizes specificity, ensuring that a positive result is virtually diagnostic of intrathecal synthesis even in the absence of OCBs. While this creates an intermediate “gray zone” (3–20), our data show that this range is effectively managed by reflex OCB testing. This combinatory approach maximizes diagnostic accuracy across the full clinical spectrum: it allows for the early exclusion of MS in patients with very low indices (< 3), reducing unnecessary further CSF investigations, while providing a safety net for borderline cases where OCB positivity remains the deciding factor. By rounding the threshold to 20, we offer a transferable and pragmatic tool that simplifies decision‐making for clinicians and laboratory specialists without compromising the stringent specificity required for MS diagnosis. However, it should be noted that this 100% specificity was observed strictly in the context of differentiating MS from NIND. While a threshold > 20 serves as a powerful rule‐in tool, its generalizability to a broader differential diagnosis—including inflammatory mimics—requires caution, as some inflammatory conditions may also exceed this value. These findings align with and reinforce growing evidence from the literature supporting the diagnostic value of the KFLC‐index (Agnello et al. 2020; Marlas et al. 2022). Across recent large cohorts, diagnostic thresholds for the KFLC‐index typically range from 6 to 10, balancing optimal sensitivity and specificity, although significant heterogeneity exists depending on methodology and patient selection (Leurs et al. 2020; Gastaldi et al. 2017; Gurtner et al. 2018; Levraut, Laurent‐Chabalier, et al. 2023). Selecting a higher cut‐off to avoid OCBs research in isoelectrofocalisation, such as > 20 as proposed by Vecchio et al. (Vecchio et al. 2024), diverges from most prior studies but can be justified in settings where maximizing specificity is needed—such as when the pretest probability for MS is low or when avoiding false positives is paramount (Leurs et al. 2020; Vecchio et al. 2024; Monreal et al. 2023). Our findings show that applying a high threshold leads to exceptional specificity (over 90%) while maintaining acceptable sensitivity, which might be particularly valuable to streamline diagnosis and minimize unnecessary OCB testing in ambiguous or atypical cases.

Nevertheless, the use of such a high cut‐off must be carefully contextualized: several studies highlight that lowering the threshold typically increases sensitivity, which is critical for the early identification and management of OCB‐negative or pediatric onset MS cases. Meta‐analyses and recent consensus recommendations generally endorse moderate thresholds (around 6–7), noting that the risk of delayed diagnosis with very high cut‐offs could result in missed opportunities for early intervention (Hegen, Walde, et al. 2023; Hegen, Arrambide, et al. 2023). Therefore, while our algorithm supports integrating high KFLC‐index values for rapid rule‐in of MS, local validation remains essential to ensure diagnostic reproducibility and to balance specificity with the need for timely treatment initiation in varied clinical scenarios.

The optimal Youden‐derived thresholds identified in our cohorts (20.27 and 10.82) highlight the biological heterogeneity and real‐world variability of MS populations. Interestingly, the replication cohort was notably younger than the derivation cohort, both for MS patients (median 31 vs. 40 years) and NIND controls (55 vs. 61 years). This younger demographic profile was associated with a lower median KFLC‐index in MS patients (45.9 vs. 64.9) compared to the derivation cohort. Such a shift suggests that the replication cohort may capture patients at an even earlier stage of the disease spectrum. This is further supported by the clinical phenotypes: the replication cohort was primarily composed of RRMS (78.8% vs. 64.5%) with a very low prevalence of progressive forms (6.1% vs. 16.1%). In these earlier stages, including RIS and CIS cases (15.1% of the replication cohort), the intrathecal inflammatory signal is often more subtle. This overall reduction in both biological baseline (in younger NIND controls) and inflammatory signal (in early‐stage MS) explains why the optimal threshold shifted downward to 10.82 in the replication group. Conversely, the higher threshold of 20.27 in the older derivation cohort underscores the need for increased stringency as age and disease duration progress. Despite this variability, our three‐tier algorithm (3–20) remains robust: it is sensitive enough to capture early‐stage patients with lower indexes while maintaining the absolute specificity required in older populations through reflexive OCB testing.

These findings provide a robust, independent confirmation of the results previously reported by Vecchio et al. (2024) and Hegen et al. (2025). In the current landscape, where various thresholds have been proposed (ranging from 6 to 20), achieving such concordance across independent cohorts is essential to provide clinicians with reliable, reproducible tools. Our data demonstrate that while a high KFLC‐index (> 20) provides immediate diagnostic confirmation, systematic OCB testing in the 3–20 range acts as a critical safety net, preserving maximal sensitivity regardless of the patient's age or disease stage.

Our proposed diagnostic strategy, based on KFLC‐index thresholds alongside OCBs, aligns with this multimodal framework and provides a flexible approach (Figure 4B):

- A KFLC‐index < 3 suggests a low probability of MS and may avoid further CSF workup;
- A KFLC‐index > 20 is highly predictive of MS and supports early clinical decision‐making;
- Intermediate values, between 3 and 20, can be resolved with systematic OCBs testing, preserving sensitivity in borderline cases:
  ○ If OCBs are negative and the KFLC‐index is below 10, the result remains negative for MS.
  ○ If OCBs are negative but the KFLC‐index is equal to or > 10, MS cannot be excluded.
  ○ If OCBs are positive, regardless of the KFLC‐index within this intermediate range, the probability of MS is high.

As proposed by Hegen et al. (2025) or Rosenstein et al. (2021), we identified a “gray zone” between KFLC‐index values of 3 and 20, in which reflexive OCB testing is recommended. By incorporating a cut‐off value of 10 into the diagnostic algorithm (corresponding to the measured 10.82 rounded down to the nearest whole number), we further refine and enhance the diagnostic precision of the proposed tool (Figure 4B).

Several limitations warrant consideration. First, both the derivation and replication cohorts were recruited at a single center and analyzed using the same assay platform (Optilite, The Binding Site). While this monocentric design ensures analytical consistency and reflects real‐world diagnostic workflows, it inevitably constrains the generalizability of our findings. Inter‐assay and inter‐platform variability in free light chain measurement are well‐documented, and the thresholds proposed herein may not directly translate across laboratories using distinct platforms, reagents, or calibration protocols. Although our results were validated prospectively in an independent cohort, broader multicenter studies employing harmonized procedures remain essential to confirm clinical applicability and facilitate inter‐laboratory comparisons (Leurs et al. 2020; Levraut, Laurent‐Chabalier, et al. 2023). Although obtained in a single‐center study, our results are generalizable and corroborate the findings of Hegen et al. (2025). Second, the control population primarily consisted of patients with non‐inflammatory neurological disorders rather than healthy volunteers. This approach mirrors routine diagnostic settings, where CSF analysis is indicated due to neurological symptoms suggestive of MS or alternative pathology. However, it may lead to an overestimation of specificity, as diagnostically challenging inflammatory mimics are underrepresented. Recent multicenter studies argue that inclusion of other inflammatory diseases in the control group enhances diagnostic rigor and helps define more universally applicable cut‐offs (Leurs et al. 2020; Rosenstein et al. 2021). Thus, while our symptomatic, non‐inflammatory control group supports external clinical validity, future research should include a broader spectrum of controls to fully characterize diagnostic accuracy. Then, a potential limitation of our study is the exclusion of OIND from the formal ROC and Youden index analyses. At the initial stages of this prospective work, 11 OIND patients were identified (including 7 with sufficient samples for FLC concentrations measurement). These patients were excluded to maintain a clear focus on the differentiation between MS and NIND, ensuring a robust derivation of diagnostic thresholds. Consequently, the reported specificity does not fully reflect the performance of the KFLC‐index in a clinical setting where inflammatory mimics are present. Indeed, 2 out of our 7 evaluable OIND patients displayed index values > 20, suggesting that the “rule‐in” claim for this threshold should be strictly interpreted as MS versus non‐inflammatory controls rather than versus all possible neurological diagnoses. While this exclusion might theoretically impact generalizability to inflammatory MS‐mimics, a preliminary application of our proposed algorithm to these 7 OIND cases yielded otherwise reassuring results. None were misclassified as “non‐inflammatory” (index < 3). Five patients fell into the intermediate range (3–20) and were OCB‐positive, correctly triggering further clinical evaluation as per our protocol. The remaining two patients had an index > 20; although high, their clinical presentations were distinct from MS, and the algorithm's role as a “rule‐in” tool remained clinically coherent. These observations align with recent literature. Vecchio et al. (2024) demonstrated that while KFLC concentrations are elevated in OIND (such as NMOSD or MOGAD), they remain significantly lower than those observed in MS, with thresholds ≥ 20 maintaining high discriminatory power. Similarly, although KFLC‐indices can be elevated in CNS infections or HIV‐associated neuroinflammation, they rarely reach the high levels typical of MS. Furthermore, our cohorts primarily included early‐stage MS (CIS and RIS) with a limited proportion of progressive MS (≈11% overall). Given that KFLC concentrations are typically higher in relapsing–remitting forms than in progressive ones, our high‐specificity threshold remains particularly suited for early diagnostic workups where the clinical stakes are highest.

Moreover, a potential limitation of our algorithm is the use of a linear KFLC‐index instead of Reiber‐based hyperbolic models. While hyperbolic models provide a more nuanced correction for severe blood–CSF barrier dysfunction, our data show that the albumin quotient was low and stable across our cohorts (median QAlb = 0.5), suggesting that barrier breakdown was not a confounding factor. Furthermore, the two‐step nature of our algorithm, which triggers reflexive OCB testing for intermediate index values, provides an inherent safety mechanism for patients with atypical protein profiles.

Finally, our handling of values below the lower limit of quantification (LLOQ) involved assigning the LLOQ value for any measurement reported as below detection, similar to routine laboratory practice. Alternative approaches—such as imputing half the LLOQ as described by Levraut, Laurent‐Chabalier, et al. (2023)—introduce further nuances. While such methodological choices potentially impact precision, our emphasis on elevated KFLC‐index values for diagnostic decision‐making likely mitigates their influence on clinical outcomes. Nonetheless, standardization of imputation strategies will be important for future cross‐study comparisons.

## 5 Conclusion

The KFLC‐index has emerged as a robust, quantitative biomarker for intrathecal immunoglobulin synthesis, demonstrating strong diagnostic value for multiple sclerosis (Hegen, Arrambide, et al. 2023). While its methodological advantages over oligoclonal bands are clear, it is important to recognize that no standardized strategy has yet been established for its optimal integration into routine CSF diagnostic workflows. Various frameworks have been proposed, ranging from systematic assessment of both KFLC‐index and OCBs to using the KFLC‐index as an initial screening tool with follow‐up OCB testing reserved for borderline or inconclusive cases (Vecchio et al. 2024; Monreal et al. 2023; Hegen et al. 2025; Morello et al. 2024). Against this heterogeneous backdrop, our validation of a simple, threshold‐based algorithm in an independent cohort of symptomatic patients directly supports practical clinical application and aligns with the real‐world diagnostic context reflected in the 2024 McDonald criteria (Deisenhammer et al. 2025; Levraut et al. 2025).

Notably, our findings—obtained entirely independently and using a single standardized reagent‐platform combination—are remarkably concordant with those reported by both Vecchio et al. (2024 and Hegen et al. 2025), despite differences in patient populations and study design. This high level of reproducibility underscores the reliability of the KFLC‐index as a decision marker. At the same time, our study reinforces the critical importance of local threshold validation, given the well‐documented inter‐assay variability in serum and CSF free light chain measurement, which may influence absolute KFLC‐index values across laboratories. Rather than diminishing generalizability, this highlights the need for harmonization of analytical procedures and multicenter benchmarking efforts to enable the broadest clinical utility.

## Author Contributions

**Noé Verchere:** data curation, formal analysis, visualization, project administration, writing – original draft, writing – review and editing, investigation, validation. **Ségolène Roult:** conceptualization, methodology, data curation, investigation, validation, formal analysis, writing – original draft. **Laure Michel:** conceptualization, methodology, software, resources, writing – review and editing, investigation. **Emmanuelle Le Page:** conceptualization, methodology, software, resources, writing – review and editing, investigation. **Chloé Rousseau:** methodology, validation, project administration, writing – review and editing. **Khaled Messaoudi:** conceptualization, methodology, resources, validation, writing – original draft, writing – review and editing. **Riwan Hasbini:** investigation, formal analysis, validation, writing – review and editing, resources. **Claude Bendavid:** conceptualization, methodology, validation, resources, writing – original draft, writing – review and editing. **Erwan Dumontet:** conceptualization, methodology, project administration, data curation, validation, formal analysis, writing – original draft, writing – review and editing. **Caroline Moreau:** conceptualization, methodology, project administration, visualization, investigation, data curation, validation, formal analysis, writing – original draft, writing – review and editing.

## Supporting information


**Table S1:** Description of MS patients in derivation and replication cohorts.
**Table S2:** Description of Non‐inflammatory neurological disorders controls in derivation and replication cohorts.

## Data Availability

The data that support the findings of this study are available from the corresponding author upon reasonable request.
