# Supplementary material for: The Kappa Free Light Chains Index in Multiple Sclerosis: From Emerging Evidence to Clinical Application
Source: J Neurochem. 2026 Apr 1;170(4):e70423. doi: 10.1111/jnc.70423 (PMC13040513; doi:10.1111/jnc.70423)
Supplement: Supplementary file 1 — Table S1: Description of MS patients in derivation and replication cohorts. Table S2: Description of Non‐inflammatory neurological disorders controls in derivation and replication cohorts. [file JNC-170-e70423-s001.docx]

**Supplementary data**

**Table S1.** Description of MS patients in derivation and replication cohorts

| **Parameter** | **Derivation cohort**  **(n = 31)** | **Replication cohort**  **(n = 33)** |
| --- | --- | --- |
| Age. years (median [IQR]) | 40 [25–48] | 32 [28–44] |
| Female sex. n (%) | 24 (77.4%) | 24 (72.7%) |
| RRMS n (%) | 20 (64.5%) | 26 (78.8%) |
| Progressive MS n (%) | 5 (16.1%) | 2 (6.1%) |
| CIS n (%) | 1 (3.2%) | 1 (3.0%) |
| RIS n (%) | 5 (16.1%) | 4 (12.1%) |
| CSF protein (g/L) | 0.4 [0.3–0.5] | 0.4 [0.3–0.4] |
| Serum protein (g/L) | 70 [67–73] | 70 [68–73] |
| CSF albumin (mg/L) | 234.0 [176–286] | 230.7 [197.8–283.6] |
| Serum albumin (g/L) | 41.5 [38.6–43.0] | 46.2 [43.7–48.3] |
| Albumin quotient (QAlb) | 0.5 [0.4–0.7] | 0.5 [0.4–0.6] |
| Blood-CSF barrier dysfunction. n (%) | 6 (19.4%) | 6 (18.2%) |
| CSF IgG (mg/L) | 47.0 [30.0–64.0] | 37.0 [28.0–58.0] |
| Serum IgG (g/L) | 10.6 [9.7–14.0] | 11.7 [10.3–13.0] |
| IgG-index | 0.8 [0.5–1.0] | 0.6 [0.5–1.1] |
| OCBs positive. n (%) | 28 (90.3%) | 30 (90.9%) |
| CSF KFLC concentration (mg/L) | 4.3 [1.1–9.1] | 3.3 [1.3–4.1] |
| Serum KFLC concentration (mg/L) | 12.2 [9.4–13.8] | 10.9 [10.1–14.6] |
| KFLC-index | 64.9 [25.6–149.4] | 45.9 [21.0–89.4] |
| CSF LFLC concentration (mg/L) | 1.2 [0.9–2.4] | 1.6 [1.1–2.0] |
| Serum LFLC concentration (mg/L) | 11.9 [8.8–14.8] | 11.2 [9.5–12.5] |
| LFLC-index | 22.0 [12.6–53.2] | 30.3 [20.5–43.5] |

Abbreviations: CIS: Clinically Isolated Syndrome; CSF: Cerebrospinal Fluid; IgG: Immunoglobulin G; IQR: Interquartile Range; KFLC: Kappa Free Light Chains; LFLC: Lambda Free Light Chains; MS: Multiple Sclerosis; n: number; OCBs: Oligoclonal Bands; QAlb: Albumin Quotient; RIS: Radiologically Isolated Syndrome; RRMS: Relapsing-Remitting Multiple Sclerosis

**Table S2.** Description of Non-inflammatory neurological disorders controls in derivation and replication cohorts

| **Parameter** | **Derivation cohort**  **(n = 49)** | **Replication cohort**  **(n = 37)** |
| --- | --- | --- |
| Age. years (median [IQR]) | 61 [39–69] | 55 [47–60] |
| Female sex. n (%) | 34 (69.4%) | 22 (59.5%) |
| CSF protein (g/L) | 0.3 [0.3–0.4] | 0.4 [0.3–0.4] |
| Diagnosis Category |  |  |
| *Neurodegenerative (Alzheimer, Parkinson...)* | 23 (46.9%) | 17 (46.0%) |
| *Vascular (stroke, TIA)* | 5 (10.2%) | 6 (16.2%) |
| *Epilepsy* | 3 (6.1%) | 7 (18.9%) |
| *Psychiatric* | 2 (4.1%) | 2 (5.4%) |
| *Other* | 16 (32.7%) | 5 (13.5 %) |
| Serum protein (g/L) | 69 [66–73] | 67 [65–71] |
| CSF albumin (mg/L) | 220 [164–258] | 219.5 [190.1–270.2] |
| Serum albumin (g/L) | 41.8 [40.0–44.4] | 44.3 [42.1–46.3] |
| Albumin quotient (QAlb) | 0.5 [0.4–0.7] | 0.6 [0.4–0.6] |
| Blood-CSF barrier dysfunction. n (%) | 0 (0.0%) | 0 (0.0%) |
| CSF IgG (mg/L) | 25.0 [18.0–31.0] | 21.0 [17.0–29.0] |
| Serum IgG (g/L) | 10.6 [8.9–12.8] | 10.0 [8.5–11.3] |
| IgG-index | 0.4 [0.4–0.5] | 0.4 [0.4–0.5] |
| OCBs positive. n (%) | 0 (0.0%) | 1 (2.7%) |
| CSF KFLC concentration (mg/L) | 0.3 [0.3–0.3] | 0.3 [0.3–0.3] |
| Serum KFLC concentration (mg/L) | 13.1 [10.3–16.3] | 11.6 [10.4–16.2] |
| KFLC-index | 3.9 [2.9–6.2] | 4.9 [3.2–6.5] |
| CSF LFLC concentration (mg/L) | 0.7 [0.7–0.8] | 0.9 [0.8–1.0] |
| Serum LFLC concentration (mg/L) | 12.7 [10.0–15.9] | 10.6 [8.6–14.6] |
| LFLC-index | 10.1 [7.7–20.9] | 18.2 [12.3–23.8] |

Abbreviations: CSF: Cerebrospinal Fluid; IgG: Immunoglobulin G; IQR: Interquartile Range; KFLC: Kappa Free Light Chains; LFLC: Lambda Free Light Chains; n: number; NIND: Non-Inflammatory Neurologic Disorders; OCBs: Oligoclonal Bands; QAlb: Albumin Quotient; TIA: Transient Ischemic Attack
